# Treatment of obstructive uropathy in one of three young brothers suffering from Gorlin-Cohen syndrome: a case report

**DOI:** 10.1186/1471-2490-12-2

**Published:** 2012-01-10

**Authors:** Ioannis Vakalopoulos, Spyridon Kampantais, Panagiotis Dimopoulos, Christos Papastavros, Vasileios Katsikas

**Affiliations:** 1 A Urologic Department of Aristotle University of Thessaloniki, "G.Gennimatas" General Hospital, 41 Ethnikis Aminis Str, Thessaloniki 54635, Greece

## Abstract

**Background:**

Frontometaphyseal dysplasia, or Gorlin-Cohen syndrome, is an X-linked disorder primarily characterized by skeletal dysplasia, such as hyperostosis of the skull and abnormalities of tubular bone modeling. Some patients develop extraskeletal manifestations, such as urinary tract anomalies.

**Case presentation:**

A 26-year-old male patient was diagnosed with frontometaphyseal dysplasia and suffered from chronic urine retention. Although the patient was primarily diagnosed with a neurogenic bladder, our work-up revealed posterior urethral valves, bladder neck stenosis, and multiple bladder stones. The patient was treated by transurethral resection of the urethral valves and bladder neck with simultaneous open cystolithotomy to remove the bladder calculi. After removal of the catheter, the patient voided normally and had no post-void residual urine. At the 1-year follow-up, he was still voiding normally; his urodynamic investigation was also normal.

**Conclusions:**

In the recent literature, there is scarce information on the diagnosis, treatment, and follow-up of patients with malformations of the urinary tract as a result of Gorlin-Cohen syndrome. The case presented here could guide urological approaches to patients suffering from this rare condition.

## Background

Frontometaphyseal dysplasia, also called Gorlin-Cohen syndrome, is a rare hereditary X-linked syndrome initially described in 1969. It encompasses cranial hyperostosis, abnormal tubulation of cylindrical bones, and other skeletal and extraskeletal abnormalities [1]. Obstructive uropathy is a feature of the syndrome in some patients [2]. Although several authors have commented on the presence of this urological complication, no specific evaluation of the symptoms or their treatment has been reported. We herein describe a male patient with typical frontometaphyseal dysplasia and the approach for treatment of his urinary tract malformations.

## Case presentation

A 26-year-old male patient who had been diagnosed with neurogenic bladder outlet obstruction and bladder stones was referred to our department for treatment. The patient was the youngest of three brothers aged 26, 28, and 30 years. Although the parents were normal, all three boys were homozygotes for frontometaphyseal dysplasia. The patient was unable to void spontaneously; therefore, for the previous 5 years, he carried a permanent urethral catheter with a stopcock mechanism to allow him to empty his bladder when he had a voiding sensation. The oldest brother was still able to empty his bladder by intermittent catheterization, and the middle brother was able to void voluntarily with low flow.

The patient claimed "normal" voiding until the age of 16 years, although he never experienced a truly "normal" urination. Because of renal colic at that age, he had an ultrasound scan in which bilateral moderate hydronephrosis and thickening of the bladder walls were observed. However, no further investigation or treatment of these symptoms was performed. Five years ago, the patient was urgently admitted because of urinary retention. Severe bilateral ureterohydronephrosis appeared on the ultrasound scan. A Foley catheter was inserted, and he then drained urine through the catheter.

Upon admission, the patient brought results of a recent urodynamic investigation that included results from urethral profilometry and voiding cystometry. Voiding cystometry during filling revealed normal sensation, capacity, and compliance of the bladder and the absence of detrusor overactivity. Although he had a strong voiding sensation with 500 mL, there was no increase in detrusor pressure suggesting urination, and the patient was unable to void and thus undergo a uroflowmetry test. The conclusion of the urodynamic investigation was a hypoactive bladder along with urethral obstruction.

The patient had the typical appearance and bone radiographic findings of Gorlin-Cohen syndrome. Characteristic features were present, including sclerotic supraorbital ridges, skull hyperostosis, micrognathia with prominent mandibular antegonial notch, scoliosis, and elongation of the metacarpals and phalanges.

**Figure 1:**
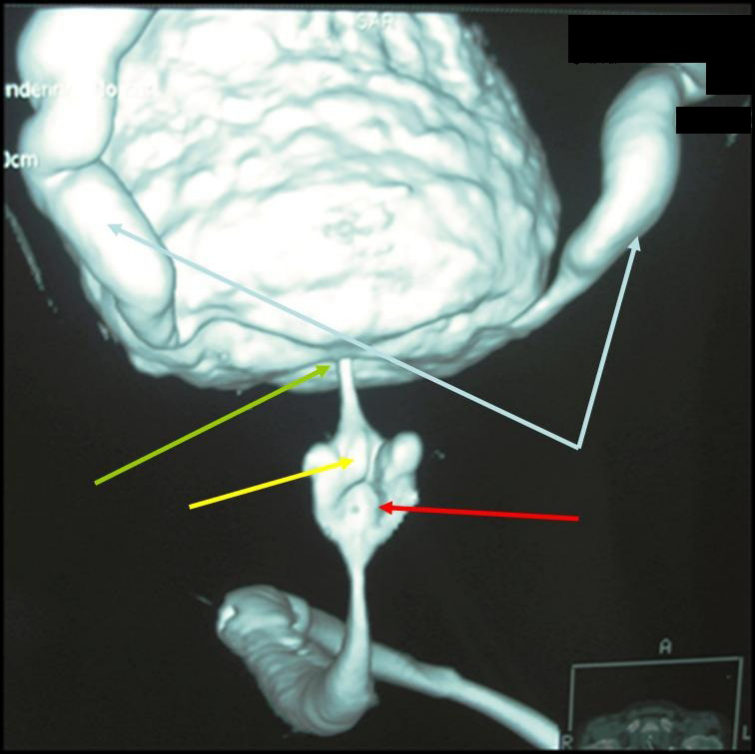
**Three-dimensional computed tomography image**. Three-dimensional computed tomography scan of a retrograde cystourethrogram reveals dilatation of the prostatic urethra (yellow arrow), a hypertrophic verumontanum (red arrow), bladder neck stenosis (green arrow), bladder distension with wall thickening, and vesicoureteral reflux with moderate ureter dilatation (blue arrows).

**Figure 2:**
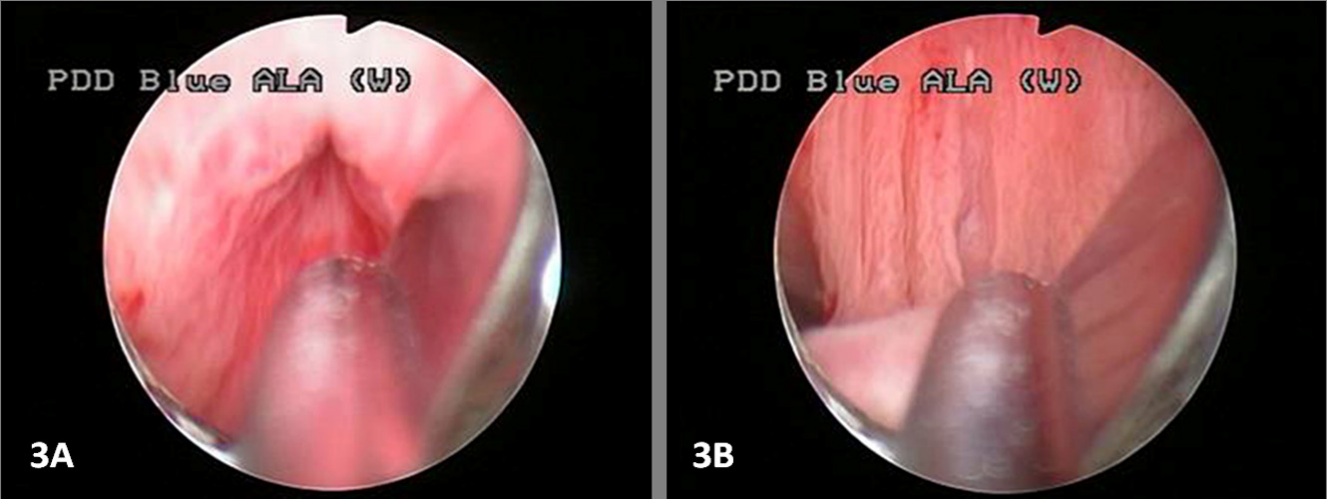
**Urethroscopy**. **(A) **Severe bladder neck contracture. **(B) **Prostatic urethra with longitudinal mucosal folds between the verumontanum and bladder neck.

Laboratory investigations were normal, and an ultrasound scan revealed some degree of residual ureterohydronephrosis and multiple stones in the bladder. Three-dimensional computed tomography of a retrograde cystourethrogram revealed dilatation of the prostatic urethra, a hypertrophic verumontanum, bladder neck stenosis, bladder distension with wall thickening, and vesicoureteral reflux with moderate ureterohydronephrosis (Figure 1). Finally, cystourethroscopy revealed a normal penile and membranous urethra, but a dilated prostatic urethra with longitudinal mucosal folds between a hypertrophic verumontanum and bladder neck, which probably represented the least common type (type 2) of urethral valves and severe bladder neck contracture (Figure 2).

**Figure 3:**
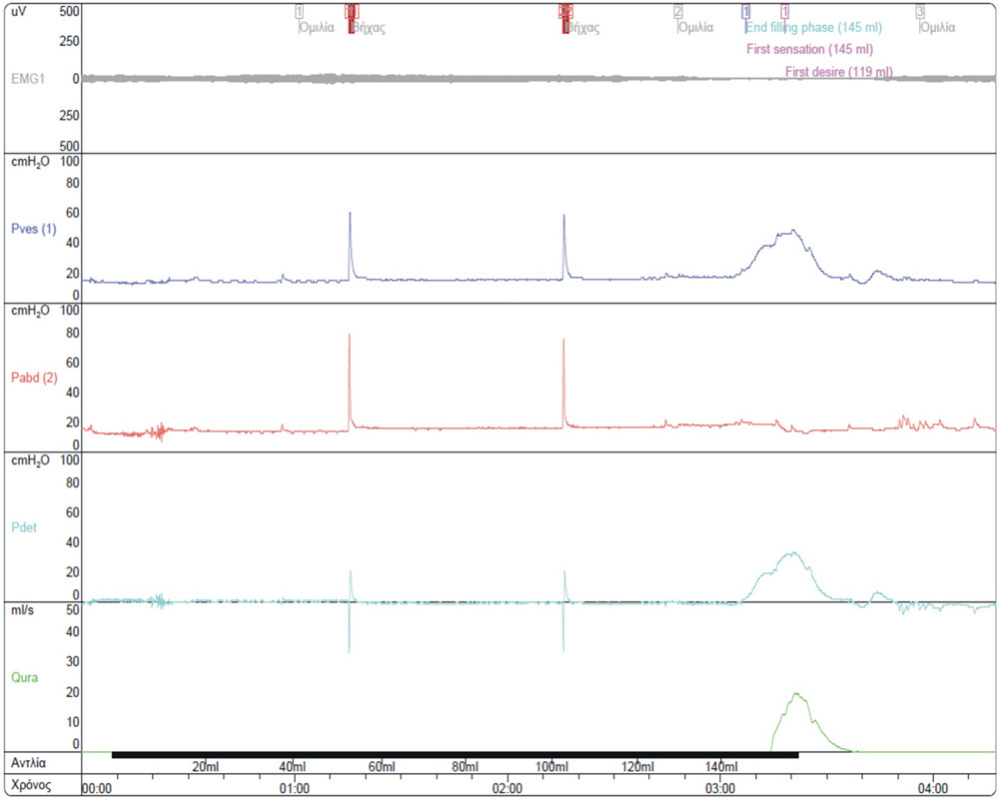
**Voiding cystometrogram**. A voiding cystometrogram performed 1 year postoperatively shows normal voiding with low pressures.

After patient consent was obtained, acknowledging that he was highly likely to experience postoperative retrograde ejaculation, he underwent urethral valve excision and bladder neck incision. Because of the difficulty of removing the bladder stones endoscopically, he also underwent an open cystolithotomy during the same operation. A total of 28 stones were removed from his bladder. The catheter was removed on the fifth postoperative day, and the patient was able to void and empty his bladder without residual urine. One year later, the patient continues to report normal micturition. The urodynamic investigation revealed normal pressure flow study results with Q_max _of 19 mL/s and P_det _in peak flow of 33 cmH_2_O. The patient voided 205 mL without a residual volume (Figure 3). Ultrasound examination of the patient revealed mild residual hydronephrosis without bladder stones.

## Discussion

Frontometaphyseal dysplasia is a rare genetic syndrome characterized by skeletal dysplasia comprising hyperostosis of the skull and modeling anomalies of tubular bones [1]. The most common manifestations include supraorbital hyperostosis, hypertelorism, down-slanting palpebral fissures, and generalized skeletal dysplasia. The dysplasia manifests with thickening of the calvarium; agenesis of the frontal, ethmoidal, and sphenoidal sinuses; and bowing and undermodeling of tubular bone diaphyses and metaphyses [3,4].

A proportion of individuals have missense mutations or small deletions in an X-linked gene, *FLNA*. The possibility of X-linked inheritance was suggested because affected individuals show some variation in clinical severity of the disease, and manifestations in females are normally milder than in males [5,6]. Despite this finding, the observation that there is partial expression in female carriers suggests that locus heterogeneity may exist for this disorder [7].

In addition to the obvious bony changes, patients have extraskeletal manifestations, which include upper airway malformations and recurrent respiratory symptoms, conductive hearing loss, underdeveloped musculature, mental retardation, cardiac defects, and genitourinary malformations [7].

Our case is typical of this syndrome with the exception of the urological manifestations, which are only briefly described in the literature. To date, of the few dozen male cases that have been reported worldwide, only approximately 32% [7] have had some form of congenital obstructive uropathy [2]. Although the most common urologic complication of the syndrome is urethral obstruction [7], there is not a meticulous description of the symptoms or their treatment.

However, on reviewing the literature, an obstructive uropathy has been previously reported. A 10-year-old male patient, briefly described by Sauvegrain et al., presented with bilateral dilatation of the renal pelvises and ureters. Although the authors did not comment on the urethra, this anomaly suggests the presence of a lower urinary tract obstructive lesion [4]. One patient, reported by Kassner et al., died from septic pyelonephritis due to hydronephrosis [8]. The child described by Kanemura et al. had stenosis of the external urethral meatus, stricture of the membranous urethra, stenosis of the right ureterovesical junction, and bilateral hydronephrosis with hydroureters [9]. Fitztimmons et al. also reported a male patient suffering from frontometaphyseal dysplasia. From the time of delivery, the patient suffered from posterior urethral valves and bilateral hydroureters with hydronephrosis. The valves were successfully treated by diathermy [2]. An 11-year-old boy with frontometaphyseal dysplasia was described by Lee et al. as having bilaterally urinary tract malformation and chronic urinary tract infections. To improve his facial appearance, this patient's prominent supraorbital ridges were contoured by ostectomy. A urologic operation was performed simultaneously [10]. Morava et al. described another male patient diagnosed with frontometaphyseal dysplasia who had recurrent hematuria. Cystourogram results suggested the presence of a mild constriction at the proximal urethra, which required treatment with antibiotics [11].

This study indicates that obstructive lesions of the ureters and urethra commonly accompany frontometaphyseal dysplasia and should be specifically sought and excluded in individuals for whom the diagnosis is being considered. Our case represents a guide to how patients suffering from this rare syndrome may be approached to assess and correct functional and anatomical anomalies.

Before presentation to our department, the patient underwent a urodynamic evaluation elsewhere. From these results, our colleagues concluded that his main problem was neurogenic bladder dysfunction. They attributed this to his rare genetic syndrome, although Gorlin-Cohen syndrome has never been previously reported to be accompanied by neurological manifestations, and the patient appeared mentally and neurologically normal. Their suggestion was for the patient to undergo a permanent vesicostomy by the Mitrofanoff procedure to allow emptying of his bladder by self-catheterizations; urethral self-catheterizations were very difficult because of his anatomic malformations. After meticulous radiologic and endoscopic examination, this diagnosis proved to be incorrect, and the patient only required minor surgery to successfully treat his severe outflow obstruction.

## Conclusions

Patients with Gorlin-Cohen syndrome deserve special care in diagnosis and treatment of their urological manifestations. A thorough urogenital search should be conducted at the time of delivery and in the first years of life to prevent major kidney complications. With synchronous cooperation with other specialties, it should be possible to improve both the outcome and quality of life of these patients.

## Consent

Written informed consent was obtained from the patient for publication of this case report and accompanying images. A copy of the written consent is available for review by the Editor-in-Chief of this journal.

## Competing interests

The authors declare that they have no competing interests.

## Authors' contributions

IV drafted the report, cared for the patient, and approved the final version of the manuscript. SK, PD, and CP performed the clinical follow-up and contributed to data acquisition. VK participated in the study design and coordination. All authors read and approved the final manuscript.

## Pre-publication history

The pre-publication history for this paper can be accessed here:

http://www.biomedcentral.com/1471-2490/12/2/prepub
